# Niosomal Nanocarriers for Enhanced Skin Delivery of Quercetin with Functions of Anti-Tyrosinase and Antioxidant

**DOI:** 10.3390/molecules24122322

**Published:** 2019-06-24

**Authors:** Banyi Lu, Yanting Huang, Zhongyun Chen, Jingyi Ye, Hongyu Xu, Wenrong Chen, Xiaoying Long

**Affiliations:** 1 School of Traditional Chinese Medicine, Guangdong Pharmaceutical University, Guangzhou 510006, China; lby0607@163.com (B.L.); hyt9601@163.com (Y.H.); hongyx0663@163.com (H.X.); 2 Department of Pharmaceutics, School of Pharmacy, Guangdong Pharmaceutical University, Guangzhou 510006, China; johnchan1995@163.com (Z.C.); small.yjy@163.com (J.Y.); 3 Research and Development Center, Sirio Pharma Co., Ltd, Shantou 515041, China; 4 Guangdong Engineering & Technology Research Center of Topical Precise Drug Delivery System, Guangzhou 510006, China

**Keywords:** anti-tyrosinase, antioxidant, niosomes, photostability, transdermal delivery

## Abstract

This study aimed to screen an effective flavonoid with promising whitening and antioxidant capacities, and design flavonoid-loaded niosomes to improve its solubility, stability, and penetration. In vitro anti-tyrosinase and 1,1-diphenyl-2-picrylhydrazyl (DPPH) free radical scavenging experiments were conducted to investigate the whitening and antioxidant capacities of several flavonoids, including quercetin, morin, festin, myricetin, rutin, and breviscapine. The conductivity, viscosity, and particle size of Span60-RH40-based formulation of nonionic surfactant vesicles (niosomes) with different mass ratios were studied to determine the most appropriate formulation. Drug-loaded niosomes were characterized for size, zeta potential, morphology, and entrapment efficiency. The photostability, solubility, release behavior, ex vivo drug penetration, and skin retention were also studied. The results showed that quercetin has considerable whitening and antioxidant capacities and Span60-RH40 at a mass ratio of 9:11 forms spherical or oval niosomes of 97.6 ± 3.1 nm with a zeta potential range of 31.1 ± 0.9 mV, and drug entrapment efficiency as high as 87.3 ± 1.6%. Niosomes remarkably improved the solubility and photostability of quercetin. Furthermore, compared to quercetin solution, quercetin-niosomes had the advantages of sustained release and improved transdermal penetration, with skin retention 2.95 times higher than quercetin solution.

## 1. Introduction

Flavonoids are widely distributed in fruits, vegetables, and herbs, and have extensive biological activities, including antiviral, anti-inflammatory, anticancer, antioxidant, and anti-tyrosinase [1,2]. Recently, the search for a safe new tyrosinase inhibitor has received much attention in the field of cosmetics, because some of the well-known inhibitors, such as kojic acid, hydroquinone, and corticosteroids, may trigger health problems, such as dermatitis and skin irritation, ochronosis, cytotoxicity, and skin cancer [3]. Due to its low toxicity and its considerable antioxidant and anti-tyrosinase activities, flavonoids have attracted great interest as potential natural whitening and antioxidant agents for use in skin care products [4,5,6]. However, flavonoids have many different structures, including different configurations with the same mother nucleus, but it is still unclear which kinds of structures possess both potent anti-tyrosinase and antioxidant capacities. In addition, most flavonoids have poor water solubility and liposolubility, and moderate lipophilicity and low molecular weight are factors known to promote better penetration. Another significant factor influencing the effectiveness of components is the biological barriers of the skin itself, such as the stratum corneum. Although physicochemical methods, including iontophoresis, ultrasound, microneedle, non-needle injection systems, and penetration enhancers, are able to promote the penetration of active ingredients through the stratum corneum, these methods can be invasive, irritate the skin, and are expensive. On the other hand, some novel carrier-mediated active ingredients delivery systems have emerged, including emulsion, micelles, vesicles, and cyclodextrin, to enhance the efficacy of existing ingredients, especially those with poor solubility and bioavailability. Vesicles have received more attention for transdermal delivery. Among vesicles, transfersomes are well-deformable liposomes with enhanced percutaneous permeability and can improve drug solubility and stability. Transfersomes are composed of a specific ratio of phospholipids and surfactants, whereas phospholipids are expensive and easy to oxidize. Therefore, searching for other stable and replaceable vesicles is an interesting research object. Niosomes are non-ionic surfactant vesicular systems, first reported in 1979 when applied in the cosmetics industry [7], but have since been studied as a drug delivery system in pharmaceutics [8,9,10,11,12,13]. The physical properties, preparation techniques, and structures of niosomes are highly similar to those of liposomes. However, niosomes also have advantages in transdermal drug delivery, such as sustained drug release, better penetration and higher skin retention [14], are less expensive to prepare, and are more stable [15]. Therefore, niosomes can be applied to the cosmetics industry [16,17,18].

The early surfactant vesicles were prepared using ionic surfactants [19,20,21,22]. The comprehensive toxicity studies on surfactants have indicated that cationic surfactants are the most toxic, followed by anionic surfactants, whereas non-ionic surfactants are the least toxic. Hence, taking safety and industrial application into consideration, the use of niosomes comprising non-ionic surfactants is a better option for transdermal delivery. Due to the structure of the amphiphilic bilayers, a Hydrophilic–Lipophilic Balance (HLB) scale range of 4 to 8 will be easier to form niosomes from [14,23,24]. Therefore, formulated, non-ionic surfactants with different HLB value will be easier for niosomes formation. Span, Tween, and Brij are common non-ionic surfactants used to prepare niosomes [15,25,26,27,28,29,30,31,32,33,34]. Non-ionic surfactants with long hydrocarbon chains without double bonds are eligible candidates because stable niosomes with the potential for increased drug loading can be formed. Span60, with a low HLB value, has been widely used to form niosomes [15,29,31,33,34]. Non-ionic surfactants with high HLB values, such as Tween20, Tween60, Tween80, Brij35, and Brij58, do not easily form niosomes on their own or without an optimal amount of cholesterol [28,35,36], because of their bulky hydrophilic heads and short hydrocarbon chains. Therefore, they are only able to form niosomes with the addition of cholesterol or when formulated with other surfactants [10,27,31,37,38,39]. Cremophor RH40 is highly hydrophilic and has low toxicity, and is widely used in formulations of oral drugs, but no reports have indicated the use of Cremophor RH40 in the formulation of niosomes. 

In this study, we screened for flavonoid with good whitening and antioxidant effects and investigated Span60-RH40, a non-ionic surfactant system with different lipid ratios to form niosomes. Quercetin loaded niosomes were characterized for vesicle size, zeta potential, encapsulation efficiency, and transmission electron microscopy (TEM), and its solubility and photostability were also investigated. In addition, rats were used to investigate the transdermal penetration of quercetin-loaded niosomes.

## 2. Results

### 2.1. Tyrosinase Inhibition of Flavonoids

**Figure 1:**
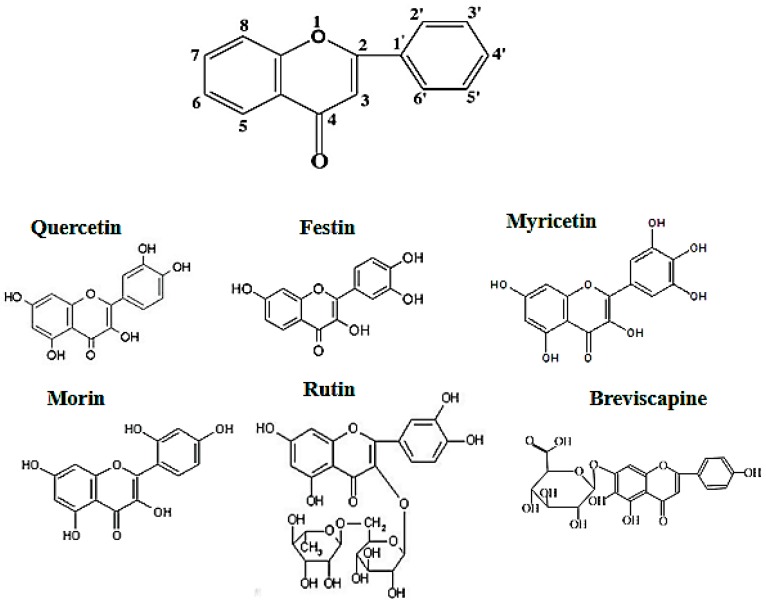
Flavonoid structure.

It is well known that flavonoids possess multiple hydroxyl groups (Figure 1) that are important to anti-tyrosinase activity. It is reported that the number and position of hydroxyl groups as well as the presence of sugar moieties are related to tyrosinase inhibition. In Table 1, Myricetin showed no the anti-tyrosinase activity because it easily forms strong intermolecular hydrogen bonds that may hinder chelation with tyrosinase. Rutin and breviscapine showed no promising anti-tyrosinase activity, whereas quercetin, morin, and festin exhibited considerably more anti-tyrosinase activity than arbutin, which is a common whitening agent used in cosmetics. 

### 2.2. Antioxidant Activity of Flavonoids

Flavonoids are able to scavenge free radicals as antioxidants by donating a hydrogen atom. The antioxidant activity of flavonoids is related to the number of hydroxyl groups and their structure. This includes: (i) the presence of an ortho-hydroxyl group in the B ring; (ii) the presence of a C2-C3 double bond in the C ring; (iii) or the presence of a C-3 hydroxyl group [40,41,42]. As shown in Table 2, the 1,1-diphenyl-2-picrylhydrazyl (DPPH) radical scavenging activity of the six compounds is in the follow order: quercetin = festin > myricetin = morin > rutin > BHT ≈ breviscapine. Quercetin and festin had the most potent radical scavenging capacity.

### 2.3. Formulation of Niosomes

#### 2.3.1. Conductivity of the Span60-RH40 System

Transfersomes are unstable because they are primarily composed of phospholipids that can be easily oxidized, causing drug leakage. Niosomes were identified as an alternative for transfersomes in transdermal delivery. Although many non-ionic surfactants are able to form niosomes [15,26,28], non-ionic surfactants with unsaturated hydrocarbon chains are less stable than non-ionic surfactants with saturated hydrocarbon chains [37]. Therefore, in this study, we chose Span60 and RH40, both of which do not possess double bonds, to form niosomes in order to improve its stability.

**Figure 2:**
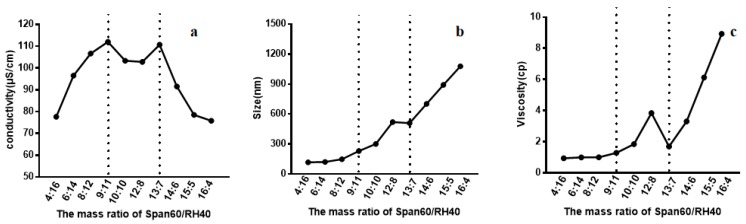
(**a**) Conductivity, (**b**) size, (**c**) and viscosity of the Span60-RH40 system in different mass ratios.

**Figure 3:**
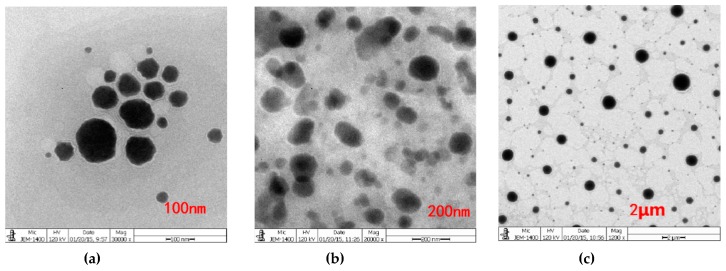
Transmission electron microscopy (TEM) images of the Span60-RH40-system in the mass ratio of at 9:11 with different scales of (**a**) 100 nm, (**b**) 200 nm, (**c**) 2 μm. Niosomes were formed.

Conductivity measurements were conducted to determine the aggregation behavior of non-ionic surfactant in aqueous solution. The conductivity of Span60-RH40 dispersed in water increased gradually from a mass ratio of 4:16 to 9:11, then plateaued between the mass ratios of 9:11 to 13:7 and decreased from 13:7 to 16:4 (Figure 2a). There were two breakpoints that may suggest changes of microstructure of the aggregates. As showed in Figure 3, at the mass ratio of 9:11, niosomes began to be formed.

#### 2.3.2. Size of the Span60-RH40 System

Particle size is the important index of the evaluation of nanometer particles. Measuring the size of Span60-RH40 system with different mass ratios of surfactant could further reflect the change of the microstructure of aggregates in the system. As shown in Figure 2b, initially, the aggregate size was relatively small and constant at the mass ratio of 4:16 to 9:11. Afterward, the aggregate size increased greatly at the mass ratio of 9:11 to 13:7, and then continually increased sharply at the mass ratio of 13:7 to 16:4, which was even up to ~1 μm. The spontaneous structural transition of Span60/RH40 aggregates was further confirmed by the aggregate size combined with the conductivity and viscosity. 

#### 2.3.3. Viscosity of the Span60-RH40 System

The viscosity of Span60-RH40 system shown in Figure 2c presented corresponding changes. Firstly, the viscosity of Span60-RH40 system was low and constant at the mass ratio of 4:16 to 9:11. Then, at the mass ratio of 9:11 to 13:7, the viscosity changed significantly, with the viscosity increasing and afterward decreasing. The conductivity and size discussed above also occurred corresponding with changes at this mass ratio range of Span60-RH40. Subsequently, at the mass ratio of 13:7 to 16:4, the viscosity sharply increased. 

### 2.4. Size, Zeta Potential, Morphology and Entrapment Efficiency of Quercetin-Niosomes

Quercetin was loaded by Span60-RH40 niosomes and then homogenized with a 1500 bar. As shown by Table 3, the hydrodynamic diameter of blank niosomes and quercetin-niosomes was 146.4 ± 5.4 nm and 97.6 ± 3.1 nm, respectively.

**Figure 4:**
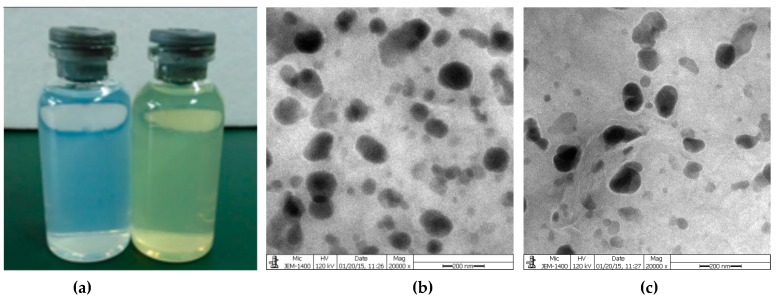
(**a**) Appearance of empty niosomes and quercetin-niosomes; (**b**) Transmission electron microscopy (TEM) image of blank niosomes; (**c**) TEM image of quercetin-niosomes.

Zeta potential was studied to understand the surface charges of niosomes. The zeta potential of blank niosomes and Quercetin-niosomes was −41.2 ± 0.6 mV and −31.1 ± 0.9 mV, respectively (Table 3). The TEM image in Figure 4b showed that quercetin-niosomes were spherical or oval in shape. The entrapment efficiency of quercetin-niosomes was 87.3 ± 1.6%, which was higher than those in the range of 48.4% to 78.4% in previous works [37,43,44]. The increased drug entrapment efficiency may be attributed to the affinity between drugs and the bilayers of niosomes [45]. 

### 2.5. Water Solubility and Equilibrium Solubility of Quercetin

**Figure 5:**
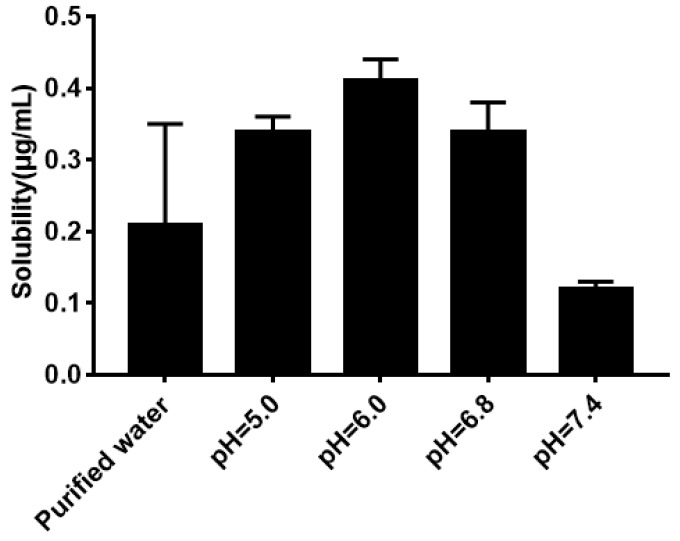
Solubility of quercetin in water and phosphate buffers.

**Figure 6:**
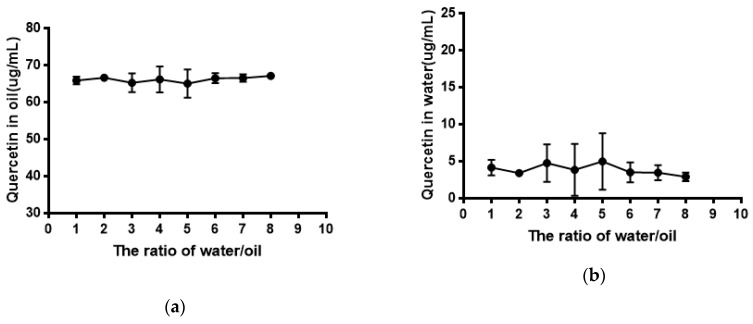
(**a**) Quercetin in oil; (**b**) Quercetin in water.

**Figure 7:**
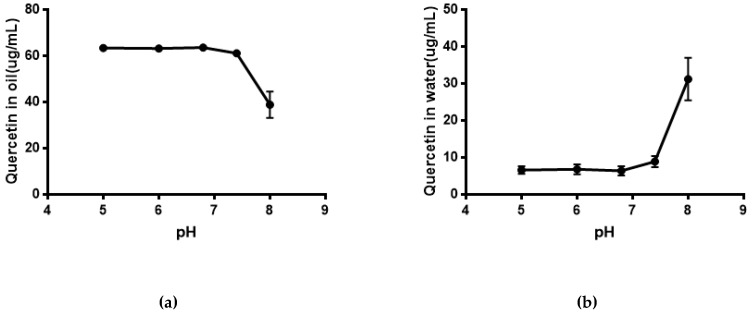
(**a**) Quercetin in oil at different pH; (**b**) Quercetin in water at different pH.

As shown in Figure 5, the solubility of quercetin in water was extremely low. Its solubility had no significant difference (*P* > 0.05) in phosphate buffer with different pH compared with that in water. The result of the equilibrium solubility of quercetin showed that quercetin was a highly liposoluble component. As seen in Figure 6 and Figure 7, the equilibrium solubility of quercetin remained stable in different ratios of water/*n*-octanol but decreased at pH 8.0 because the structure of quercetin changed under basic conditions, causing the increase in water solubility. However, the poor water solubility of quercetin was limited to its application. Based on Table 4, the solubility of quercetin improved in surfactants discussed below, and especially in 0.6% Tween80 (*P* < 0.05). However, its solubility was further improved by loading into niosomes. Quercetin (300 μg/mL) was loaded in niosomes and the high encapsulation of Span60-RH40 niosome was 87.3 ± 1.6%, therefore, the solubility of quercetin in niosomes was 261.9 ± 4.8 μg/mL, which was 1247-fold higher than that in water (*P* < 0.05).

### 2.6. Photostability of Quercetin-Niosomes

**Figure 8:**
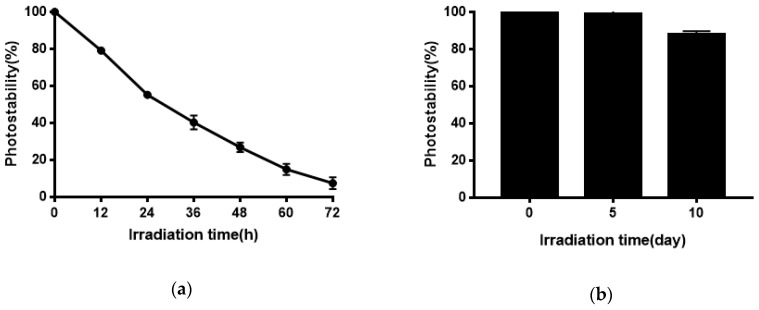
(**a**) Photostability of Quercetin solution; (**b**) the photostability of Quercetin-niosomes.

Quercetin has poor photostability and quickly degrades under exposure to sunlight or UV irradiation [46,47,48], possibly because quercetin undergoes photooxidation upon UV irradiation [49]. However, photostability is necessary for quercetin to show its activities. As shown in Figure 8, niosomes effectively improved the photostability of Quercetion and remained 88.06% after 10 days of exposure to strong illumination. Quercetion was protected from UV irradiation because it was encapsulated in the bilayers of niosomes.

### 2.7. In Vitro Release Behavior of Quercetin-Niosomes

The appropriate release medium was screened to maintain sink conditions, therefore the solubility of quercetin in the different mediums was assessed. The results in Table 5 demonstrate that quercetin had a higher solubility in Tween80 aqueous and its solubility increased gradually with increasing concentration of Tween80. The solubility of quercetin increased to 76.07 ± 3.11 μg/mL in acidic conditions. Therefore, considering the pH of human skin, 0.8% Tween80 (pH 5.0) was chosen as the release medium to study the release behavior of free quercetin and quercetin-niosomes.

**Figure 9:**
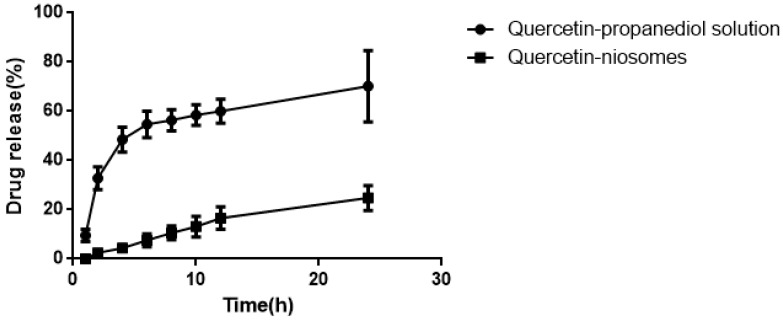
Release behavior of quercetin-propanediol solution and quercetin-niosome in 0.8% Tween80 (pH 5.0).

As seen in Figure 9, compared to quercetin-niosomes, free quercetin released much faster in the release medium, demonstrating that niosomes had a sustained release effect. Sustained release reduces application time, reduces toxicity, and maintains effective drug concentration.

### 2.8. Ex Vivo Permeation and Skin Retention Studies

A 0.6% Tween80 solution was used as the penetration release medium in this study. In our ex vivo penetration study, quercetin could not be determined in the penetration release medium, which illustrated that quercetin did not penetrate the dermis and reach the release medium, but niosomes remarkably improved the skin retention of quercetin (*P* < 0.05). As showed in Table 6, the skin retention of quercetin-niosomes (1.92 ± 0.74 μg/cm^2^), quercetin-niosome-1% propanediol (2.34 ± 0.40 μg/cm^2^), and quercetin-transfersomes (2.53 ± 0.40 μg/cm^2^) was significantly higher compared to that of Quercetin-propanediol (0.65 ± 0.10 μg/cm^2^) (*P* < 0.05).

## 3. Discussion

Previous studies have determined that the free hydroxyl group at C-3 position played an important role in tyrosinase inhibition [50,51,52]. Conversely, 3-*O*-glycosides may hinder their binding to the active sites of tyrosinase and may be the reason why rutin showed no anti-tyrosinase activity. In contrast, the sugar moiety located at the C-7 position had little effect on anti-tyrosinase activity and did not block chelation with tyrosinase [51]. However, the presence of an additional hydroxyl group at the C-3′ position of the B ring increased the tyrosinase inhibition [53,54]. Breviscapine did not exhibit anti-tyrosinase activity because it lacked the C-3 hydroxyl group and C-3′ hydroxyl group that may have had an effect on anti-tyrosinase activity rather than the presence of 7-*O*-glycosides. In analyzing the structure–activity relationship of quercetin and morin, we observed that the IC_50_ value of morin is about 1/3 that of quercetin and the only difference between the structure of quercetin and morin is the position of hydroxyl groups in the B ring. Therefore, the presence of *o*-dihydroxyl groups in the B ring increased anti-tyrosinase activity. The IC_50_ value of festin was about 1/20 that of quercetin and the only difference between the structure of quercetin and festin is that festin is the absence of a C-5 hydroxyl group in the A ring. The hydroxyl group at the C-5 position also plays an important role in increasing the tyrosinase inhibition activity, as it may chelate Cu^2+^ at the active site of tyrosinase with carbonyl at the C-4 position [50]. Quercetin possesses free hydroxyl groups, not only at positions C-3 and C-5 of the C ring but also at the C-3′ position of the B ring. Thus, quercetin exhibits much more potent inhibition of tyrosinase compared to the other flavonoids.

Flavonoids not only have promising anti-tyrosinase activity but also have potent antioxidant activity. Quercetin has an additional C-5 hydroxyl group; however, both quercetin and festin had the same antioxidant capacity, suggesting that the C-5 hydroxyl group had little impact on radical scavenging. Myricetin possesses more hydroxyl groups in the B ring, but it did not exhibit a stronger antioxidant activity than quercetin or festin. This may be because myricetin can form strong intermolecular hydrogen bonds and lower hydrogen atom donation. Similarly, morin showed lower antioxidant capacity than quercetin and festin because of the B ring hydroxyl configuration, which illustrated that *o*-dihydroxyl in the B ring is important for increasing radical scavenging capacity. However, rutin and breviscapine had significantly lower antioxidant capacities because rutin has glycosides at the C-3 position producing steric hindrance. Breviscapine has a single hydroxyl group at the B ring and has less free hydroxyl groups. Consequently, the results in this paper indicate that the B ring hydroxyl configuration was significant for the antioxidant capacity of flavonoids, whereas the A ring had little impact on radical scavenging capacity. Additionally, blocking or removing the hydroxyl group at the C-3 position may reduce the radical scavenging capacity of flavonoids.

Span60 and RH40 are not capable of ionization. Therefore, the charges seem to originate from the interaction between the hydrophilic head groups of Span60 and RH40 forming hydrogen bonds [55], when charged aggregates form between the lipophilic Span60 and hydrophilic RH40. Therefore, the conductivity may be due to the combination of Span60 and RH40. The conductivity increased initially, possibly because the addition of Span60, which associates with RH40, forms charged mixed micelles. Increasing Span60 also increased the number of charged aggregates and the conductivity gradually increased. A breakpoint occurred at the mass ratio of 9:11. The sudden change suggests that the microstructure of aggregates may change and niosomes may form. The conductivity subsequently slightly decreased and remained relatively constant, which may indicate the formation of niosomes, as the charges were enclosed. Another breakpoint occurred at the mass ratio of 13:7 and the conductivity decreased sharply, however, the size of the aggregates increased with the increase in Span60, possibly due to dense clusters of the larger multilamellar niosomes forming and the occurrence of white precipitate [56,57], causing the decrease of free charged aggregates. 

The spontaneous structural transition of Span60-RH40 aggregates was further confirmed by aggregate size combined with the conductivity and viscosity. When the mass fraction of RH40 was dominant, the aggregate size was relatively constant and small. The system had a water-like viscosity and mixed micelles may form. With an increasing mass fraction of Span60, the hydrophobicity of the system increased, and the viscosity and aggregate size increased. The increasing Span60 may induce the formation of niosomes. However, with a further increase in Span60, the viscosity and size also sharply increased due to the existence of abundant larger multilamellar niosomes, which caused precipitate formation. The formation of niosomes was also verified by TEM (Figure 3).

When the concentration of Span60 was low, the Span60-RH40 system had a water-like viscosity and the aggregate size was relatively small, indicative of the formation of mixed micelles. From the mass ratio of 9:11 to 13:7, the viscosity and turbidity of the system increased simultaneously. The obvious change in viscosity may be related to the transition of the micelle to a rigid and viscous noisome, as surfactants are tightly packed and bilayers form; this observation was consistent with the literature [57,58]. Between the mass ratios of 13:7 and 16:4, the viscosity increased significantly, possibly because of the existence of abundant larger multilamellar niosomes causing precipitate formation. 

Interestingly, quercetin-niosomes had smaller size than blank niosomes, which was in accordance with previously published studies [43]. It has been illustrated that if molecules showing good affinity with the constituent surfactants of niosomes were incorporated inside the bilayers, it would cause a reduction in vesicle size. This observation may due to the effect of a strong affinity for drug and niosomes on holding different lamellae together, making the membrane more rigid [14]. The absolute value of zeta potential for quercetin-niosomes was lower than that of empty niosomes (Table 3). This may be due to the interaction of quercetin with the head group of surfactants neutralizing the negative charges on the surface of niosomes. This observation was also reported previously; that drugs could reduce the negative surface charges [59].

We determined that quercetin was insoluble in water and phosphate buffer. In previous studies, to maintain a sink condition of poorly soluble drugs in penetration release medium, cosolvents, including alcohols and surfactants, are often used [14], therefore, a 0.6% Tween80 solution was used as penetration release medium to keep the sink condition. However, taking efficacy into consideration, for skin care and the treatment of skin diseases, it is more effective and safer to detain components or drugs deep in the skin than to enter into the blood circulation. In our study, although quercetin did not penetrate through dermis into medium, the skin retention experiments showed that compared to free quercetin, niosomes improved the penetration of quercetin into the epidermis. Therefore, niosomes may be a promising delivery system for cosmetic purposes and the treatment of skin diseases. Quercetin is highly liposoluble and its low water solubility has limited its development and application. Quercetin-loaded-niosomes have not only improved the water-solubility, but also the transdermal absorption of quercetin. Quercetin-niosomes had a 3-fold increase in skin retention over the quercetin-propanediol solution. After adding 1% propanediol, a penetration enhancer, into the original formulation of Span60-RH40 niosomes, its transdermal absorption improved, comparable to that of quercetin-transfersomes. 

## 4. Materials and Methods

### 4.1. Materials

Quercetin, morin, festin, myricetin, rutin, and breviscapine were purchased from DATIAN Biology Co (Shanxi, China), β-arbutin from Beijing Brilliance Biochemical Co, Ltd (Beijing, China), and 1,1-diphenyl-2-picrylhydrazyl (DPPH) from Guangzhou Qiyun Bio-technology Co (Guangzhou, China). Sodium dihydrogen phosphate, disodium hydrogen phosphate, dimethyl sulfoxide (DMSO), and absolute ethyl alcohol were purchased from DAMAO CHEMICAL REAGENT FACTORY (Tianjin, China). Span60 was purchased from Tianjin Kemiou Chemical Reagent Co, Ltd (Tianjin, China) and Cremophor RH40 from Guangzhou Jiefu Trading Co, Ltd (Guangzhou, China). Male Sprague-Dawley rats (200 ± 20 g) were used and supplied by the Guangdong Animal Experiment Center (Guangzhou, China). Rats were kept under standard temperature, humidity, and light conditions, with a standard rodent diet and water. The research was approved by Guangdong Pharmaceutical University Experimental Animal Ethics Committee (NO. SPF2017120, Guangzhou, China).

### 4.2. In Vitro Tyrosinase Inhibition Activity of Flavonoids

The tyrosinase inhibition assay was performed based on a previous study with slight modification [2]. The activity of tyrosinase inhibition was determined using a spectrophotometer by monitoring dopachrome formation at 493 nm. The reaction system, as shown in Table 7, contained 0.5 mL of different concentrations of flavonoids, including arbutin, quercetin, morin, festin, rutin, and breviscapine, and 0.5 mL of tyrosinase (120 unites/mL in phosphate buffer, pH 6.8), which were combined with 1.5 mL of phosphate buffer (pH 6.8). After incubation at 30 °C for 10 min, 0.5 mL of l-tyrosine was added to the systems. The reaction systems were incubated at 30 °C for 20 min and absorbance was determined at 493 nm. Arbutin was used as a positive control. The degree of inhibition of the sample was expressed as 50% inhibition concentration (IC_50_). 

The inhibitory effects of the test samples were expressed as the percentage of tyrosinase inhibition as follows:

(1) $$\mathrm{Tyrosinase}\;\mathrm{inhibition}=\frac{\left(A-B)-(C-D\right)}{\left(A-B\right)}\times 100\%$$

where *A* and *B* denote the absorbance at 493 nm of the mixture without the test sample (l-tyrosine mixed with the enzyme in the buffer; the control) and without the test sample or the enzyme (l-tyrosine in the buffer; the blank), respectively; *C* and *D* denote those with the test sample and enzyme (l-tyrosine mixed with the enzyme and test sample in the buffer; the reaction mixture) and without the enzyme (l-tyrosine mixed with the test sample in the buffer; the blank version of *C*).

### 4.3. In Vitro Antioxidant Activity of Flavonoids

The antioxidant activity of flavonoids was measured by the bleaching rate of DPPH, reported by Blois [60]. In its radical form, DPPH absorbs at the wavelength of 515 nm, but its absorption decreases upon reduction by an antioxidant or a radical compound. The DPPH radical scavenging activity of flavonoids was evaluated according to the method described by Rui Yang, with some modifications [61]. Briefly, 5 mL of DPPH (30 μg/mL) was mixed with 0.1 mL of absolute ethyl alcohol and 0.1 mL of samples with different concentrations. Then, the absorbance of the mixtures was determined at 515 nm. The DPPH radical scavenging activity was calculated as follows:

(2) $$\mathrm{DPPH}\;\mathrm{radical}\;\mathrm{scavenging}\;\mathrm{activity}\;=\;\frac{A_{0}-A_{i}}{A_{0}}\times 100\%$$

where *A_0_* and *A_i_* denote the absorbance at 515 nm of the control (DPPH mixed with absolute ethyl alcohol) and the test samples (DPPH mixed with flavonoids).

### 4.4. Formulation of Niosomes

Based on previous research and published papers that showed that Span60 is a common surfactant used to form niosomes [15,27,31], we designed a formulation system of non-ionic surfactants by using Span60 and RH40 at different mass ratios. The conductivity, viscosity, and size as indexes were investigated to determine the proper ratio of non-ionic surfactants forming niosomes.

The mass ratios of Span60 and RH40 investigated were 4:16, 6:14, 8:12, 9:11, 10:10, 12:8, 13:7, 14:6, 15:5, and 16:4 (total weight: 2 g). Span60 was melted at 80 °C and then mixed with RH40 by a stirrer for 2 min. Then, the mixtures were hydrated with 100 mL of water at 80 °C for 10 min, respectively. The conductivity (DDS-11A conductometer, Shanghai Ridao Scientific Instruments Co., Ltd., Shanghai, China), viscosity (Ubbelode viscometer, 0.4–0.5 mm, Shanghai Liangjing Glass Instrument Factory, Shanghai, China), and size (Nano-ZS90, Malvern, Malvern, UK) of non-ionic surfactant systems were measured at room temperature.

### 4.5. Preparation and Evaluation of Quercetin-Loaded Niosomes

#### 4.5.1. Preparation of Quercetin-Loaded Niosomes

Based on the TEM image of the Span60-RH40 system (Figure 3), Span60, and RH40 at a mass ratio of 9:11, spherical or oval niosomes appear to form. Therefore, quercetin-loaded niosomes were prepared with the Span60/RH40 mass ratio of 9:11 using a magnetic stirring apparatus. Span60 (0.9 g), RH40 (1.1 g), and quercetin (30 mg) were melted together. Then, 100 mL purified water at 80 °C was added to the mixture and hydrated for 10 min. The quercetin-loaded niosomes were formed and then cooled down to room temperature. Clear niosomes were acquired by processing with high-pressure homogenization at 1500 bar.

#### 4.5.2. Size, Zeta Potential and Morphology of Quercetin-Loaded Niosomes

The size and zeta potential of empty niosomes and quercetin-loaded niosomes were measured using Malvern Zetasizer (ZS90 + MPT-2, Malvern, Malvern, UK). The morphology of quercetin-loaded niosomes was characterized by transmission electron microscopy. The quercetin-niosomes samples were deposited on a copper grid and after 1 min, excess sample was removed with filter paper. A TEM was used to observe the samples after negative staining by phosphotungstic acid.

#### 4.5.3. Drug Entrapment Efficiency

The drug entrapment efficiency of quercetin-loaded niosomes was determined using minicolumn centrifugation technique. Minicolumn was prepared using hydrated Sephadex G50 dispersion into a 5mL syringe, which was centrifuged for 30 s at 900 rpm. An aliquot of quercetin-loaded niosomes (400 μL) was taken into the minicolimn and the elutes were collected by using centrifugation at 1500 rpm for 3 min. The elutes was diluted to 10 mL with methanol and vortexed, and 10 μL of the filtered samples were injected into high performance liquid chromatography(HPLC). Then, free quercetin was eluted by centrifugation with 25 mL 80% methanol and the content of quercetin was determined by HPLC.

### 4.6. Water Solubility and Equilibrium Solubility of Quercetin

The water solubility of quercetin was measured with 15 mL of purified water, phosphate buffers with different pH values (pH 5.0, 6.0, 6.8, 7.4, 8.0), and excess quercetin, and placed into vials. The vials were shaken for 24 h in a 37 °C water bath and the supernatant was determined by HPLC after centrifugation.

The equilibrium solubility of quercetin was performed using an *n*-octanol-water system with ratios of *n*-octanol/water of 1:1, 1:2, 1:3, 1:4, 1:5, 1:6, 1:7, and 1:8. Then, 70 μg/mL of quercetin solution was prepared by water-saturated *n*-octanol solution and 1 mL of 70 μg/mL quercetin solution was taken in eight vials and then 1 mL, 2 mL, 3 mL, 4 mL, 5 mL, 6 mL, 7 mL, and 8 mL purified water were added to respective vials. The equilibrium solubility of quercetin in *n*-octanol-buffer systems at different pH values was also studied. The ratio of *n*-octanol-buffer was chosen at 1:2 based on the result of the equilibrium solubility of quercetin in the *n*-octanol-water system. A 2 mL volume of 70 μg/mL of quercetin solution and 4 mL of *n*-octanol-saturated phosphate buffer solutions at different pH values were added to vials and the vials were shaken for 24 h in a 37 °C water bath. Then, 0.2 mL of quercetin solution in the *n*-octanol layer were taken and diluted to 5 mL (about 2.8 μg/mL) using mobile phase (methanol-1% phosphoric acid = 60:40). Samples were determined by HPLC. Standard quercetin *n*-octanol solution (70 μg/mL) was diluted and determined as described above.

### 4.7. Photostability of Quercetin-Niosomes

Quercetin methanol solution (300 μg/mL) was added to transparent vials and placed under the sunlight. Then, the content of quercetin was determined by HPLC at 0, 12, 24, 36, 48, 60, and 72 h. Quercetin-niosomes were aliquoted into transparent vials and placed inside the light stability test chamber (PTOP-500B, Zhejiang TOP Instrument Co., Ltd., Zhejiang, China) with illuminance of 4500 ± 500 LX for 10 days. A 0.2 g sample of quercetin-niosomes was taken out on days 0, 5, and 10, and dissolved in 10 mL ethanol. Samples were determined by HPLC after centrifugation and filtration with a 0.22 μm Millipore filter membrane. The photostability was evaluated by the percentage of remaining quercetin at different irradiation times, compared to unexposed samples.

### 4.8. In Vitro Release Study

#### 4.8.1. The Solubility of Quercetin in Different Release Medium

The solubility of quercetin was investigated in different release medium, including 0.1%, 0.2%, 0.3%, 0.4%, 0.6%, and 0.8% Tween80 aqueous solution, as well as 0.2%, 0.4%, and 0.6% SDS aqueous solution. An aqueous solutions of 15 mL of Tween80 and SDS at different concentrations and excess quercetin were placed into each of the vials. Vials were shaken in a 32 °C water bath for 24 h. After 24 h, 5 mL suspension was centrifuged at 3000 rmp for 15 min and 0.2 mL of the supernatant was diluted to 2 mL and determined using HPLC.

#### 4.8.2. Drug Release by Dialysis

The solubility study of quercetin presented in Table 5 illustrated that 0.8% Tween80 (pH 5.0) aqueous solution fulfilled sink conditions and corresponded to the pH value of human skin. Therefore, the release behaviors of free quercetin and quercetin-niosomes were investigated in the 0.8% Tween80 (pH 5.0) aqueous solution.

Dialysate bag (8000–14,000 molecular weight cutoff) was processed with 2% NaHCO_3_ and 1 mmol/mL EDTA solutions. A 10 mL of (300 μg/mL) Quercetin-niosomes and quercetin-propanediol solution were added into dialysate bags and were placed into dissolution vessels containing 1000 mL of 0.8% Tween80 (pH 5.0) aqueous solution at 32 °C. The dissolution vessels were placed on a magnetic stirrer at the speed of 100 rmp. The release medium (2 mL) was withdrawn at 1, 2, 4, 6, 8, 10, 12, and 24 h, and replaced with blank release medium (2 mL). The 2 mL of release medium withdrawn was centrifuged at 3000 r/min for 15 min and filtered. The released quercetin was quantified by HPLC. 

### 4.9. Ex Vivo Drug Permeation and Skin Retention Studies

Rats were sacrificed by cervical dislocation, hair on the abdomen was shaved, and then abdominal skin was separated and placed into physiological saline. Afterward, subcutaneous tissue (muscle, fat, and vascellum) was removed using surgical scissors and hemostatic forceps and then stored at 4 °C with physiological saline for further investigation.

The permeation and drug skin retention studies of quercetin-niosomes, quercetin- niosomes-1% propanediol, quercetin-transfersomes (composed of phospholipid and Tween80 at the mass ratio of 9:11), and free quercetin-propanediol solution were carried out to investigate their transdermal delivery using TK-20B transdermal diffusion device with an effective diffusion area of 2.99 cm^2^. Then, 0.6% Tween80 solution was selected as the penetration release medium. Skin tissues were placed between the diffusion cell and receptor cell, and 0.4 mL of quercetin preparations (equivalent to 120 μg of quercetin) was applied on the skin. Penetration medium (0.5 mL) was withdrawn from the receptor cell at 1, 2, 4, 6, 8, 10, 12, and 24 h with the replacement of fresh penetration medium (0.5 mL). Penetration mediums withdrawn at different timepoints were diluted to 7 mL with methanol and determined by HPLC after centrifugation and filtration. 

Subsequent to the permeation experiment, drug skin retention experimentation was performed. After 24 h, skin tissues were isolated, then sheared completely in 7 mL methanol after washing off quercetin on the surface using distilled water and extracted for 40 min ultrasonically. Then, samples were vortexed and centrifuged, and the supernatant was analyzed using HPLC after filtration through 0.22 μm Millipore filter membrane.

### 4.10. HPLC Method

The analytical column was Dimark C18 (150 mm × 4.6, 5 μm). Mobile phase was composed of methanol and 1% H_3_PO_4_ (60:40, *v*/*v*). The flow rate was set to 1 mL/min at 30 °C and an injection volume of 10 μL. The quercetin peak was detected at 363 nm.

### 4.11. Statistical Analysis

Data are expressed as the mean of experiments ± the standard deviation (SD) and were analyzed using one-way analysis of variance (ANOVA), followed by Tukey’s multiple comparison post-test. Statistical differences yielding *P* < 0.05 are considered statistically significant.

## 5. Conclusions

We screened quercetin, a natural flavonoid possessing potent whitening and antioxidant capacities, and formulated niosomes using a nonionic surfactant system to improve the solubility, photostability, and skin penetration ability of quercetin.

## Figures and Tables

**Table 1:** Anti-tyrosinase activity of arbutin and flavonoids.

Component	IC_50_ (μg/mL) ^1^
Arbutin	98.14 ± 1.45
Quercetin	1.59 ± 0.38
Morin	4.26 ± 0.47
Festin	23.10 ± 0.32
Rutin	>1000
Breviscapine	>1000
Myricetin	—

Note: **^1^** 50% inhibitory concentration (IC_50_).

**Table 2:** 1,1-diphenyl-2-picrylhydrazyl (PPH) radical scavenging activity of flavonoids.

Component	EC_50_ (μg/mL) ^1^
Butylated hydroxytoluene (BHT)	358.15 ± 1.43
Quercetin	80.75 ± 0.26
Festin	83.19 ± 0.33
Myricetin	118.63 ± 0.57
Morin	123.06 ± 0.48
Rutin	188.28 ± 0.68
Breviscapine	368.08 ± 1.04

Note: **^1^** 50% effective concentration (EC_50_).

**Table 3:** Size and zeta potential of empty niosomes and quercetin-niosomes.

Sample	Size (nm)	Zeta Potential (mV)
Empty niosomes	146.4 ± 5.4	−41.2 ± 0.6
Quercetin-niosomes	97.6 ± 3.1	−31.1 ± 0.9

**Table 4:** Solubility of Quercetin in water, surfactants, and niosomes.

Medium	Solubility (μg/mL)
Water	0.21 ± 0.14
0.6% Tween80	56.35 ± 1.42 *
0.6% Sodium dodecyl sulfate(SDS)	6.48 ± 0.44 *
Niosomes	261.9 ± 4.8 *

Note: * *P* < 0.05 compared to water.

**Table 5:** Solubilities of Quercetin in different release medium.

Tween80 Aqueous Solution (μg/mL)		pH-0.8%Tween80 Aqueous Solution (μg/mL)		SDS Aqueous Solution (μg/mL)	
0.1%	12.19 ± 1.05	pH 5.0	79.49 ± 0.41	0.2%	4.58 ± 0.04
0.2%	24.72 ± 2.15				
0.3%	31.37 ± 0.73	pH 6.8	52.36 ± 0.71	0.4%	4.22 ± 0.43
0.4%	41.34 ± 0.19				
0.6%	56.35 ± 1.42	pH 7.4	55.02 ± 6.60	0.6%	6.48 ± 0.44
0.8%	76.07 ± 3.11				

**Table 6:** Skin retention of free quercetin and quercetin-vesicles.

Quercetin Formulation	Skin Retention (μg/cm^2^)
Quercetin-propanediol	0.65 ± 0.10
Quercetin-niosomes	1.92 ± 0.74 *
Quercetin-niosome-1%propanediol Quercetin-transfersomes	2.34 ± 0.40 * 2.53 ± 0.40 *

Note: * *P* < 0.05 compared to quercetin-propanediol solution.

**Table 7:** The reaction systems of anti-tyrosinase assay.

Reaction System	Tube Label and Volume of Reaction Solution			
	A	B	C	D
Phosphate buffer (mL)	1.7	2.2	1.5	2
Tyrosinase solution (mL)	0.5	0	0.5	0
Arbutin solution/Flavonoid solution (mL)	0	0	0.5	0.5
l-tyrosine (mL)	0.5	0.5	0.5	0.5
Dimethyl sulfoxide (DMSO) (mL)	0.3	0.3	-	-

Note: Flavonoid samples (0.5 mL) and arbutin solutions (0.5 mL) contained 0.3 mL DMSO.
